# Gender Representation in Orthopaedic Surgery: A Geospatial Analysis From 2015 to 2022

**DOI:** 10.7759/cureus.27305

**Published:** 2022-07-26

**Authors:** Nicholas J Peterman, Bailey Macinnis, Katy Stauffer, Rachel Mann, Eunhae G Yeo, Kristine Carpenter

**Affiliations:** 1 Medical School, Carle Foundation Hospital, Urbana, USA; 2 Family Medicine, Carle Illinois College of Medicine, Urbana, USA; 3 Family Medicine, Carle Foundation Hospital, Urbana, USA

**Keywords:** trailblazer, orthopaedic surgery, gender representation, gender diversity, female

## Abstract

Introduction

The gender disparity in orthopaedic surgery is well-established. According to our analysis, only 7.4% of practicing orthopaedic surgeons in the US are female in 2022. While there are several theories attempting to explain this gender gap, our eight years of data show that limited female representation is a self-perpetuating cycle as areas without female representation almost never improve in that regard. It appears that existing female mentorship is critical to the growth of a female orthopaedic presence in an area. In the present work, we aim to describe how gender diversity in orthopaedic surgery differs across the country, how this diversity is changing over time, and how surgeon gender diversity may be affected by the sociodemographic characteristics making up the counties where orthopaedic surgery is practiced.

Methods

A retrospective study was conducted using publicly available National Provider Identifier (NPI) data from 2015 to 2022. Orthopaedic surgeons and their genders were identified using the Provider Type and Gender data elements associated with an individual NPI. Rural-urban and metro characters were defined using the USDA Economic Research Reserve’s rural-urban continuum codes. Python was used for database building and data cleaning. GeoDa, a statistical map-based graphing software, was used to plot and assess demographic, geographic, and socioeconomic trends. Trends in gender diversity from 2015 to 2019 were analyzed for each individual year as well as the time period as an aggregate. Cluster analysis was performed to assess complex spatial patterns of variables that could not be condensed linearly or logarithmically. Moran’s I was used to measure the similarity of a Federal Information Processing System (FIPS) area code to its neighbors. Within the clustering analysis, spatial clusters were broken down into four groups of spatial outliers (High-High, High-Low, Low-High, and Low-Low) referencing a given area’s relationship with its neighbors. Factorial ANOVA between each of the four cluster types was performed using the variables provided in the article to identify significant demographic variables within the cluster analysis.

Results

There are relative hotspots of gender diversity in the Northwest, Northeast, and Southwest with relative coldspots in the Midwest and Southern US. In counties that are considered gender diversity hotspots, the total population of orthopaedic surgeons increases by 0.94 each year while the population of female orthopaedic surgeons increases by 0.2, suggesting that in areas with high gender diversity, 4.7 male orthopaedic surgeons are joining practices for every 1.0 female. In areas with low gender diversity, the population of orthopaedic surgeons increases by 0.11 surgeons each year while the slope for an increase in female orthopaedic surgeons is 0.

Conclusions

Orthopaedic surgery lags behind other male-dominated surgical specialties in gender parity. Our analysis demonstrates that certain areas of the country including the Northwest, Northeast, and Arizona have improved gender diversity compared to the rest of the country. We also see that the rate of increase of female orthopaedic surgeons in the past seven years is highest in areas with more preexisting female orthopaedic surgeons, suggesting the importance of a “trailblazer” phenomenon in recruiting female surgeons.

## Introduction

While medicine in general has reached gender parity in recent years, orthopaedic surgery remains far behind [1]. In 2020, females made up 16% of orthopaedic surgery residents and just 6% of practicing orthopaedic surgeons [2]. Compare this to general surgery in 2020, where females made up 42% of general surgery residents [3,4]. The gender gap for orthopaedic surgery is also closing at a slower rate than other male-dominated specialties [2]. Between 2012 to 2020, the number of female residents in plastic surgery increased by 12%, while the number of female residents in orthopaedic surgery only increased by 3.3% [3].

Some explanations for the poor female representation in orthopaedic surgery include limited exposure to the field, few female mentors, concerns over work-life balance, and stereotypes about the types of personalities that gravitate toward orthopaedic surgery [5-10]. As a result, there have been initiatives including pipeline programs, targeted mentoring, and advising for females and underrepresented minorities (URM) [8,11]. While these initiatives may be effective, especially in the immediate vicinity where they are employed, the data show that there is still much work to be done before gender parity is achieved in orthopaedic surgery [8,11]. 

In this study, we sought to identify geographic trends in the distribution of female orthopaedic surgeons. We propose that identifying geospatial pockets of greater gender diversity among orthopaedic surgeons may help uncover the key factors that increase the recruitment and retention of female surgeons. This knowledge will be invaluable in guiding future efforts to promote women in orthopaedic surgery. 

Note on language: In this paper, we have elected to use the term "female" to describe our target population, as this is the term used in our source database. We are using this term to include biological sex and/or self-identified gender and thus intend it to be synonymous with the broader term “women.” 

## Materials and methods

A retrospective study was conducted utilizing the publicly available National Plan and Provider Enumeration System (NPPES) National Provider Identifier (NPI) registry coupled with county-level United States (US) socioeconomic data from the US Census Bureau [12,13]. The full NPPES Data Dissemination file is sorted by unique NPI numbers and lists all active providers, their specialties, location of practice, and gender. This file is updated monthly but historical files are not maintained. The Wayback Machine, an internet archive, was used to download 14 total NPI dissemination files at approximately six-month intervals between 2015 and 2022 [14]. Using Python, these lists were filtered to contain only orthopaedic surgeons and then grouped by geographic county of practice. Both the number of female orthopaedic surgeons as well as the total number of orthopaedic surgeons were recorded for each county at each time point. To accurately portray the level of gender diversity in each county, a simple "percent female" would be insufficient as this would bias the data towards extremes of representation. For example, rural counties with a single female orthopedic surgeon would seem misleadingly diverse. A new metric named the "gender diversity index" was therefore created. This index represents the odds, scaled between 0 and 100, that two randomly chosen orthopaedic surgeons in a county would be of a different gender. In other words, the closer this index is to 100, the closer the gender ratio is 1:1. The equation for this metric is displayed below:

$Gender \, Diversity\, Index = 200 \times (1 -((\frac{Percent \, Female}{100})^{2} +(\frac{Percent\, Male}{100})^{2}))$

To assess for changes over time in the number of female orthopaedic surgeons, the total number of orthopaedic surgeons, and the gender diversity index, a linear regression was conducted for each variable across 14 time points for each individual county. The latest available socioeconomic and demographic data from the US Census Bureau from 2015-2019 were then also added to the dataset, on a county level. 

GeoDa (https://geodacenter.github.io/), a statistical map-based graphing software, was used for map plotting and cluster identification with Moran’s Index (Moran’s I) analysis [15,16]. The Moran I statistic was used to identify if a county and surrounding counties are statistically significantly different (p < 0.05) than the national average for a metric of interest, which in this case was the average orthopaedic gender diversity index. If both a county and its surrounding counties are significantly higher or lower than average, the county as a whole is classified as one of four types: High-High, Low-Low, Low-High, and High-Low. The first label ("High" or "Low") represents a county’s relationship to the national average, while the second attribute represents its average neighbor’s relationship to the national average. High-High and Low-Low classifications can be thought of as "hotspots" and "coldspots" of gender diversity respectively. The Low-High and High-Low classes represent areas where there is a significant contrast in gender diversity between adjacent counties. A factorial ANOVA between each of the four cluster types was performed across all county-level variables to identify statistically significant differences in the socioeconomic and demographic groups most impacted by national variation in orthopedic gender diversity. A two-tailed t-test was also conducted between the High-High and Low-Low groups alone to identify focused differences between the areas of highest and lowest gender diversity. 

## Results

**Figure 1:**
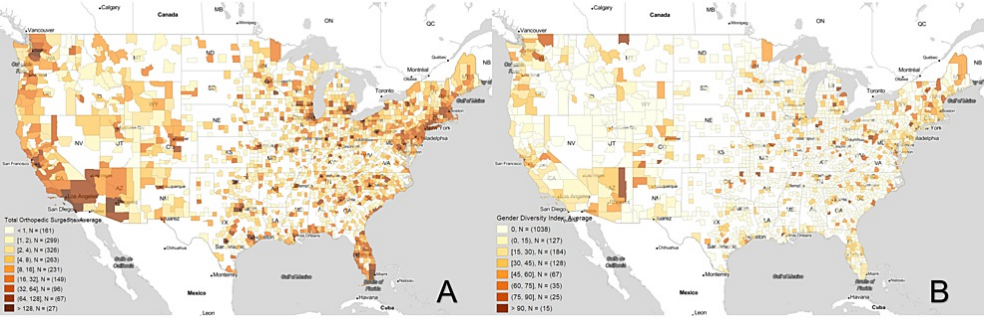
Boxplot Maps of the Average B) Total number of Orthopaedic Surgeons per County and A) Orthopaedic Gender Diversity Index from 2015 to 2022 White areas of the map contained no orthopaedic surgeons at any point in time and were excluded from the analysis.

Figure 1A shows the average number of orthopaedic surgeons per county during the years of 2015-2022 while Figure 1B demonstrates the average gender diversity index during the same time period. In some city centers in the Northwest, Northeast, and Southwest, counties with more orthopaedic surgeons tend to have greater gender diversity. There are, however, notable exceptions to this trend. Figure 1A shows that the Gulf coast areas, including Florida, have relatively high concentrations of orthopaedic surgeons. However, Figure 1B demonstrates a relative lack of gender diversity in these same areas. Similarly, orthopaedic surgeons are well-dispersed across the Midwest and Southern states, areas where Figure 1B demonstrates a significant lack of gender diversity.

**Figure 2:**
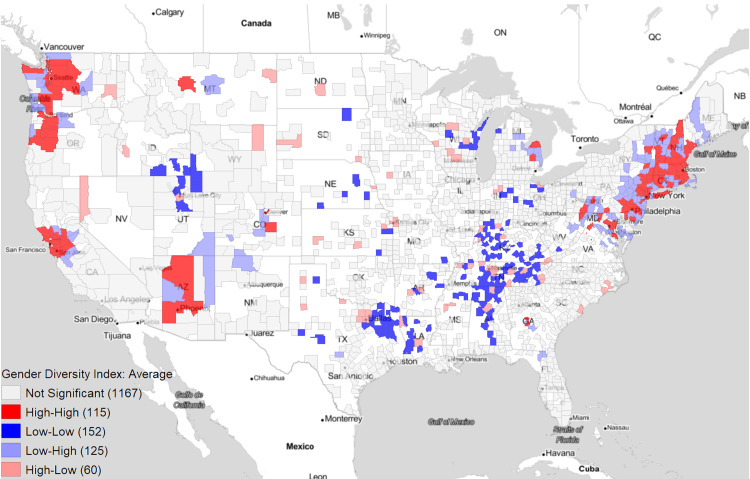
Moran’s I Cluster Analysis of the Average Orthopaedic Gender Diversity Index from 2015 to 2022 White areas of the map contained no orthopaedic surgeons at any point in time and were excluded from the analysis.

Figure 2 serves as a statistical confirmation of the above trends in gender diversity using Moran's I Cluster Analysis. We see relative hotspots for gender diversity in the Northwest, Northeast, and Southwest, with relative coldspots for gender diversity in the Midwest and Southern US.

Table 1 displays ANOVA and t-test results for the Moran’s I clustering of specific gender diversity related metrics. In this instance, ANOVA compares the four groups (High-High, High-Low, Low-High, and Low-Low) generated by Moran’s I clustering while the t-tests compare just High-High and Low-Low counties. We see a significant difference in the gender diversity index (35.69±19.58 vs 0.37±1.81, p<0.01) and female orthopaedic surgeons (4.47±7.32 vs 0.01±0.07 female orthopaedic surgeons, p<0.01) when comparing High-High counties to Low-Low counties. As demonstrated subjectively on the heatmaps, areas with high gender diversity also tend to have higher total populations of orthopaedic surgeons (46.8±62.57vs 5.01±6.64, p<0.01).

**Table 1:** Comparison of Orthopaedic Surgeon Demographics of Moran’s I Gender Diversity Index Groupings

Moran’s I Average Gender Diversity Index Groupings	High-High		Low-Low		Low-High		High-Low		P Value	
Number of Counties	115		152		125		60		ANOVA	T-TEST (H-H to L-L)
	Mean	Standard Deviation	Mean	Standard Deviation	Mean	Standard Deviation	Mean	Standard Deviation		
Gender Diversity Index										
Average	35.69	19.58	0.37	1.81	1.16	2.9	35.89	21.98	9.05E-172	2.01E-145
Slope	1.25	4.15	0.16	1	0.23	1.12	0.91	5.43	0.011611	0.002034
Pearson Coef (R2)	0.21	0.62	0.02	0.16	0.06	0.27	0.11	0.69		
Female Orthopaedic Surgeons										
Average	4.47	7.32	0.01	0.07	0.14	0.5	2.61	2.96	1.37E-164	6.18E-141
Slope	0.2	0.38	0	0.03	0.01	0.07	0.17	0.36	9.10E-13	1.08E-09
Pearson Coef (R2)	0.35	0.52	0.02	0.16	0.06	0.27	0.29	0.53		
Total Orthopaedic Surgeons										
Average	46.8	62.57	5.01	6.64	9.98	19.26	34.55	39.65	6.60E-45	1.32E-37
Slope	0.94	1.96	0.11	0.33	0.24	0.62	1.01	1.64	9.70E-10	5.87E-07
Pearson Coef (R2)	0.54	0.54	0.18	0.58	0.23	0.58	0.42	0.59		

In addition to understanding the current landscape of gender diversity in orthopaedics, we aimed to identify how this landscape is changing over time. A linear regression was calculated for each county. Slope and R2 values were averaged across Moran’s I groupings and are listed in Table 1. We see that in High-High counties, the total population of orthopaedic surgeons increases by 0.94 each year while the population of female orthopaedic surgeons increases by 0.2. This suggests that in areas with high gender diversity, 4.7 male orthopaedic surgeons are joining practices for every 1.0 female. In areas with low gender diversity, the population of orthopaedic surgeons increases by 0.11 surgeons each year while the population of female orthopaedic surgeons increases by 0. 

Table 2 lists various socioeconomic factors which are averaged for High-High, Low-Low, High-Low, and Low-High counties. We see that areas with higher gender diversity tend to be more populous (average population 544,747±624,006 vs 71,494±75,795, p<0.01), with higher population density (2917 ± 8570 vs 133 ±145.56 people per square mile, p<0.01) and greater urban characteristics (rural-urban continuum score 2.18 ± 1.54 vs 4.52±2.12, p<0.05) than areas with low gender diversity. We see that areas with more gender diversity tend to have increased racial diversity, including lower percent white (75.59±17.11% vs 87.05±11.21, p <0.01), higher percent Asian (6.06±7.03% vs 1.05±1.08%, p<0.05), and higher percent Hispanic (14.14±11.05% vs 6.98±7.92%, p<0.01).

**Table 2:** Comparison of sociodemographic factors of Moran’s I Gender Diversity Index Groupings

Moran’s I Average Gender Diversity Index Groupings	High-High		Low-Low		Low-High		High-Low		P Value	
Number of Counties	115		152		125		60		ANOVA	T-TEST (H-H to L-L)
Average Variables per County	Mean	Standard Deviation	Mean	Standard Deviation	Mean	Standard Deviation	Mean	Standard Deviation		
Population	544747.6	634006.8	71494.4	75795.3	136496.5	193275.5	319222.6	459988.1	8.24E-34	4.89E-34
Population Density	2917.08	8570.01	133.57	145.56	469.29	1208.82	476.34	615.03	6.89E-21	5.40E-22
Rural/Urban Continuum Code	2.18	1.54	4.52	2.12	3.68	2.43	2.98	1.86	1.97E-18	2.97E-21
Percent Male	49.2	0.97	49.41	1.5	49.63	1.52	49.39	1.43	0.214515	0.34838
Median age	40.07	4.41	39.55	4.44	43.49	5.41	37.8	5.09	1.58E-14	0.335523
% Multiracial	3.72	1.7	2.08	1.01	2.68	1.3	2.7	1.21	7.30E-20	1.08E-19
% White	75.59	17.11	87.05	11.21	84.44	13.9	79.17	14.49	7.05E-12	7.60E-12
% Black	8.89	11.31	7.65	10.92	6.43	9.41	12.36	14.53	0.012175	0.062425
% American Indian	0.87	2.9	0.54	0.95	1.62	7.9	0.8	1.29	0.048765	0.048679
% Asian	6.06	7.03	1.05	1.08	2.18	2.73	2.67	2.63	5.17E-23	1.64E-22
% Asian Indian	1.44	2.1	0.21	0.32	0.57	1.14	0.57	1.07	6.14E-15	6.84E-17
% Hispanic	14.14	11.05	6.98	7.92	9.29	11.58	9.85	10.55	2.73E-10	9.98E-12
% Unemployment	3.25	0.79	2.97	0.95	3.18	0.89	2.86	0.72	0.001492	0.001588
% Employed	60.24	4.84	55.16	7.35	56.75	7.74	60.18	7.43	1.72E-09	9.16E-10
% Commute: Public Transportation	7.45	12.5	0.39	0.61	1.82	2.96	1.02	1.11	3.62E-30	3.99E-25
Mean Travel Time to Work	28.22	5.97	23.42	4.29	27.23	6.03	21.28	3.71	7.81E-21	1.63E-12
% Work: Management, Business, Science, and Arts	40.7	7.82	30.83	5.25	36.39	7.27	35.47	6.75	6.83E-29	4.92E-29
% Work: Government	15.24	4.87	14.34	3.69	16.52	5.53	14.27	5.12	0.00044	0.22475
Median Household Income	73350.85	18433.24	51325.8	12471.28	65472.81	18763.96	57088.08	12206.22	6.51E-28	2.15E-26
% With Retirement Income	21.18	4.49	21.72	3.76	24.51	4.62	19.78	5.05	2.17E-12	0.235036
% With SNAP Benefits in Past Year	11.23	5.11	12.96	5.63	11.46	5.03	10.6	3.64	0.007419	0.007382
% With Health Insurance	93.96	2.5	90.48	4.02	93.22	3.6	90.5	3.7	6.96E-21	2.05E-16
% With Public Health Insurance	36.36	7.24	38.24	9.13	39.64	8.72	33.61	8.57	3.95E-05	0.087692
% Poverty	11.72	4.68	15.86	5.95	11.94	5.05	14.27	5.19	5.21E-12	6.77E-10
% 25+ Year Old: Beyond Bachelor's Degree	14.69	6.05	7.5	3.28	11.68	5.55	10.53	4.33	8.27E-31	8.70E-31
% 25+ Year Old: Bachelor's Degree or Beyond	35.65	11.11	20.97	8.19	29.42	11.73	29.05	9.59	3.63E-29	3.31E-29
% Households: English Speaking	79.83	14.09	93.11	6.37	88.36	11.89	89.13	9.79	1.81E-23	1.00E-24
% Households: Personal Computer	91.09	3.25	85.59	5.67	88.77	5.84	89.4	4.95	1.12E-17	1.17E-18
% Households: Internet Access	84.53	5.13	75.52	7.73	81.56	7.94	80.83	7.13	4.77E-24	6.00E-24

## Discussion

Orthopaedic surgery trails behind other surgical and male-dominated specialties in closing the gap between male and female surgeons [2-4]. Through geospatial analysis, we set out to identify pockets of increased or decreased gender parity in order to deduce potential factors that could facilitate or inhibit the recruitment of female orthopaedic surgeons. 

From Figure 2 it can be seen that certain areas of the country have better male-to-female orthopaedic surgeon ratios compared to others. For example, the Northeast and West as well as the state of Arizona are hotspots for high gender diversity indexes. One probable reason for this is that these regions contain large academic centers and boast high concentrations of orthopaedic surgeons [17]. As the gender diversity index looks at the likelihood that two orthopaedic surgeons from one area are of opposite gender, a larger population of surgeons will increase these odds. Conversely, a smaller population will have the opposite effect. It is likely that some of the coldspots in the Midwest and South may have only a few orthopaedic surgeons who all happen to be of the same gender. Additionally, the high number of academic institutions also indicates the presence of numerous large residency programs. Since trainees often establish their practice in the region they received their training, the females who train in these areas may ultimately end up practicing there as well [18,19]. 

The gender diversity hotspots identified in this study align with the high female surgeon clusters found in a study by Chapman et al. [18,19]. When the US was divided into hospital referral regions, this study found that nearly half of all female orthopaedic surgeons practiced within regions that had the highest proportions of female orthopaedic surgeons [18]. These high practice regions included New England and the Pacific Coast, with relative deficiencies identified in the South and Upper North Midwest (Michigan, Illinois, Ohio, Wisconsin, and Indiana). When we examined the socioeconomic characteristics of hotspots versus coldspots in Table 2, we found that hotspots, on average, were more urban, had statistically significantly higher levels of college and advanced education degrees, and had a larger percentage of workers employed in white-collar jobs. These factors may contribute to the retainment of female surgeons within these hotspot regions. Thus, while it seems reasonable that efforts to increase the proportion of female orthopaedic surgery residents should eventually yield increases in the number of practicing female orthopaedic surgeons, it is questionable whether these efforts will be recognized in more rural areas of the country. 

Our results suggest an additional, crucial factor contributing to a higher gender diversity index: areas with relatively high numbers of female orthopaedic surgeons are increasing in gender diversity at a greater rate than areas with low representation. This is demonstrated by Table 1, which lists the slope of female orthopaedic surgeons over time. In high gender diversity index (High-High) counties, the population of female orthopaedic surgeons increases by 0.2 each year, while in low gender diversity index (Low-Low) counties it increases by roughly 0. This indicates a positive correlation between higher numbers of female surgeons with the higher recruitment of female surgeons. However, this growth is minimal when compared to the increase in the total number of orthopaedic surgeons per year, which is 0.94 and 0.11 for high and low gender diversity index counties respectively. In other words, even in high gender diversity areas, there are 4.7 male orthopaedic surgeons joining practices for every one female. Our results are consistent with those found in a recent study by Acuña et al., which found that at current growth rates, it will take over 200 years to achieve gender parity (defined as at least 36.3% female) in orthopaedic surgery [20].

There is projected to be an overall shortage of orthopaedic surgeons in the coming years as the current surgeon workforce ages and retires [21]. Our data demonstrate that females can help close this deficit. This study adds to a growing body of evidence showing that female orthopaedic leaders play important roles in recruiting and retaining female trainees [10,11,22,23]. Because orthopaedic surgery is perceived by female medical students as heavily male-dominated, female mentors play crucial roles as "trailblazers" [5,9,24]. When students are able to identify themselves with an orthopaedic surgeon, female (and other underrepresented) medical students can better picture a career in orthopaedic surgery themselves [7,25]. These trailblazers can also double as mentors and provide pertinent guidance for these underrepresented people groups. It is likely that these trailblazing females within gender diversity hotspots contributed to the observed relative increase in female recruitment. Therefore, one method of addressing the orthopaedic gender gap is to increase the number of female leaders in orthopaedic residency programs and regional orthopaedic societies, where representation is lacking [23,26,27]. Pipeline programs such as the Perry Initiative and the Nth Dimension are also necessary for achieving gender parity [6,11,25]. These programs provide females with mentoring and early exposure to orthopaedics, resulting in participants of the Perry Initiative having increased rates of matriculation into orthopaedic surgery residencies when compared with students who did not receive similarly targeted mentoring [11]. 

Gender diversity in orthopaedic surgery must be prioritized, not to meet numerical quotas but to provide better care for patients. A diverse workforce brings varied experiences and perspectives, and a critical mass of 30% within any given population is cited as the number needed to allow for recognizable influence within that population [28]. Additionally, patients often prefer to be seen by a physician who is "concordant" to them by gender, race, or disability status. It is well-established that female patients often prefer female physicians, particularly for sensitive exams, however, a recent study suggests that patients may prefer concordance with their physicians even in specialty settings such as orthopaedic clinics [29]. There is also some evidence to suggest that surgical patients have better outcomes and fewer complications when there is gender concordance between the physician and the patient [30]. At the very least, increasing the number of female orthopaedic surgeons will increase the possibility that a female patient could be seen by a female surgeon if desired. 

This study has some limitations. The most recently published sociodemographic data from the US Census Bureau was from 2019. This means that the general population data used to identify the qualities of the communities categorized by the gender diversity index groupings may not fully reflect that of the current year. Secondly, this analysis is biased towards extremes of representation in areas of low orthopaedic surgeon representation. This is especially true in more rural counties in which minimal amounts of change seemed considerable. The gender diversity index was therefore applied to address this bias, as well as a linear regression which was used to determine the change in metrics over time. An additional limitation to this study is that the results produced by our analysis are generalizations of the entire counties. Some county-level data aggregates several different hospitals together, which captures only regional-level trends rather than hospital-level trends. This may cause the interhospital differences that promote physician diversity to be overlooked. Future analysis can be done at the hospital level, which will identify pockets of gender diversity and areas for improvement at the highest level of granularity.

## Conclusions

Orthopaedic surgery has the lowest percentage of female surgeons among surgical subspecialties and lags behind other male-dominated specialties in the recruitment of females. Through geospatial analysis, we found that certain areas of the country, notably the Northeast, West Coast, and Arizona, have a more balanced ratio of male to female surgeons when compared to the South and Midwest. Despite efforts to increase the number of female orthopaedic surgeons, areas with high gender parity add an average of five male surgeons for every one female. Areas with low gender parity are not adding female surgeons at any appreciable rate. Continued efforts should focus on increasing exposure to the field for young females and especially targeting recruitment to areas with low numbers of female surgeons.
